# Regulation and Use of Cannabidiol in Croatia Compared with Selected European and International Models: From Consumer Products to Clinical Practice

**DOI:** 10.3390/jcm15156123

**Published:** 2026-08-06

**Authors:** Ana Batinic, Antonia Zecic, Zeljko Dujic, Davorka Sutlovic

**Affiliations:** 1 Pharmacy of Split-Dalmatia County, 21000 Split, Croatia; ana.batinic05@gmail.com; 2 Department of Natural and Biomedical Sciences, Faculty of Forensic Sciences, University of Split, 21000 Split, Croatia; azecic@forenzika.unist.hr; 3 Department of Integrative Physiology, School of Medicine, University of Split, 21000 Split, Croatia; zeljko.dujic@mefst.hr; 4 Faculty of Health Sciences, University of Split, 21000 Split, Croatia; 5 Department of Applied Pharmacy, School of Medicine, University of Split, 21000 Split, Croatia

**Keywords:** cannabidiol, CBD, medical cannabis, dietary supplements, novel food, regulation, European Union, Croatia, pharmacy practice, training for pharmacists

## Abstract

Cannabidiol (CBD) has attracted growing scientific and clinical interest due to its potential therapeutic applications and increasing availability across Europe. This narrative review examined the regulatory status, market availability, and clinical use of CBD in Croatia in relation to selected European Union countries and international reference models (Canada and Australia). A literature search was conducted using PubMed, Scopus, and Web of Science, complemented by official regulatory and governmental sources. The analysis included Croatia, Germany, the Netherlands, Slovenia, Italy, France, and Finland, while Canada and Australia were discussed as international reference models. Considerable differences were identified regarding the regulation of CBD-containing products, access to medical cannabis, reimbursement policies, clinical implementation, and the role of pharmacists. Germany and the Netherlands have established well-developed systems for medical cannabis use, whereas Finland represents a more restrictive regulatory model. In Croatia, CBD-containing products are widely available, but their integration into routine clinical practice remains limited. Regulatory uncertainty, insufficient professional training, and the lack of standardised clinical guidance were identified as important barriers. The findings suggest that the expansion of the consumer CBD market has generally outpaced the integration of cannabinoid-based therapies into healthcare practice across the countries included in this review. Improved professional training and clearer clinical guidance may facilitate their evidence-based implementation in clinical practice.

## 1. Introduction

Cannabidiol (CBD) was first isolated from *Cannabis sativa* L. more than eight decades ago, and its chemical structure was elucidated in 1963 by researchers led by Raphael Mechoulam. Since then, CBD has attracted increasing scientific and medical interest due to its diverse pharmacological properties and potential therapeutic applications [1,2]. Botanically, hemp and marijuana belong to the same species, *Cannabis sativa* L., but are legally distinguished according to their THC content, with hemp generally defined as cannabis containing no more than 0.3% THC on a dry-weight basis and marijuana referring to cannabis varieties exceeding this amount [3,4,5]. Δ9-Tetrahydrocannabinol (THC) is the main psychoactive component of *Cannabis sativa* L. THC primarily acts as a partial agonist at cannabinoid CB1 receptors, which are part of the endocannabinoid system that regulates mood, memory, appetite, pain perception, and motor function. Although THC binds to both CB1 and CB2 receptors, it exerts its psychoactive effects primarily through CB1 receptors, which are abundantly expressed in the central nervous system. Activation of these receptors results in euphoria, altered perception, impaired memory, and changes in motor coordination and cognition [6,7]. THC is used therapeutically in selected clinical indications because of its analgesic, antiemetic, appetite-stimulating, and antispastic effects, although psychoactive effects, including euphoria, may limit its clinical use [8]. The differing pharmacological and therapeutic properties of THC and CBD are largely explained by CBD’s low affinity for CB1 and CB2 receptors, which contributes to the absence of intoxicating effects [6,7,9,10,11]. Although scientific and clinical interest has primarily focused on Δ9-tetrahydrocannabinol (THC) and cannabidiol (CBD), *Cannabis sativa* L. produces more than 150 phytocannabinoids identified to date. Most of these compounds are present in relatively low concentrations and remain considerably less studied than THC and CBD [6,12,13]. Medical cannabis refers to standardised preparations of *Cannabis sativa* L. or its extracts used for therapeutic purposes under medical supervision. These products may contain varying concentrations of Δ9-tetrahydrocannabinol (THC) and cannabidiol (CBD), ranging from CBD-dominant formulations containing less than 1% THC to THC-rich preparations containing more than 20% THC, depending on the product composition, therapeutic indication, and national regulatory framework [14,15]. According to ClinicalTrials.gov, the world’s largest publicly accessible clinical trial registry, more than 1200 studies investigating CBD have been registered across a broad range of therapeutic areas (accessed 3 June 2026). Available evidence suggests that CBD exhibits a broad range of pharmacological activities, including anticonvulsant, antiemetic, anxiolytic, analgesic, antioxidant, anti-inflammatory and potentially cardioprotective effects [16,17,18]. Its generally favourable safety profile has further increased scientific and medical interest in its potential therapeutic applications [1,17,19,20,21,22]. Due to its high lipophilicity, CBD shows low oral bioavailability. Several formulation technologies, including lipid-based delivery systems and advanced oral formulations, have been developed to enhance CBD bioavailability and improve systemic exposure [23,24,25,26]. However, the strength and quality of evidence vary considerably across different indications, with convincing clinical evidence currently available for only a limited number of therapeutic applications [27]. Within Europe, authorised cannabinoid-based medicinal products include Epidyolex^®^ (cannabidiol) for specific seizure-related indications (with Lennox–Gastaut syndrome or Dravet syndrome) and tuberous sclerosis; Sativex^®^ (nabiximols) for selected indications such as multiple sclerosis-related spasticity, and in some countries, dronabinol-containing medicinal products or magistral preparations prescribed for specific clinical indications [14,27]. The available evidence should be interpreted with caution due to substantial heterogeneity among CBD-containing products, including differences in formulation, dosage, route of administration, and cannabinoid composition [1]. While cannabidiol (CBD) is increasingly available across Europe, its clinical application remains poorly defined, and substantial differences persist among European Union (EU) member states regarding the regulation, classification, and market accessibility of CBD-containing products [28,29]. A further regulatory challenge concerns the classification of CBD products, which may fall under different legal frameworks, including medicinal product, food, cosmetic, tobacco, or drug control legislation [30]. Interest in CBD-containing food products and dietary supplements has increased markedly in recent years, resulting in 194 applications for novel food authorisation being submitted to the European Commission since 2018 [31]. The growing diversity of cannabis products in Europe is accompanied by considerable variation in national regulatory approaches and cannabis policies [31]. Within the European Union, CBD-containing cosmetic products may be placed on the market if they comply with the safety and regulatory requirements set out in EU cosmetics legislation [29]. Despite the widespread commercial availability of CBD products across Europe, EFSA has proposed a provisional safe intake level of 0.0275 mg/kg body weight per day (approximately 2 mg/day for a 70 kg adult), highlighting ongoing uncertainties regarding CBD safety [32]. Croatia provides an interesting example of a rapidly expanding CBD market accompanied by limited clinical implementation and ongoing regulatory challenges. This review examines the regulatory status, market availability, and clinical use of CBD in Croatia in relation to the European Union, with particular emphasis on dietary supplements, cosmetic products, medicinal products, and real-world clinical practice. Particular attention is given to the discrepancy between the growing commercial availability of CBD products and their limited evidence-based integration into clinical practice. Regulatory and clinical differences between Croatia and selected European countries are discussed, together with the emerging role of pharmacists in supporting the safe and rational use of CBD-containing products. To ensure consistent terminology throughout this review, the definitions of the key cannabis- and CBD-related terms are summarised in Table 1.

## 2. Research Findings

This review manuscript presents the results for the situation in Croatia in comparison with six European countries and Canada and Australia, which were taken as an international reference model. The results are presented by country and focus on the regulatory framework, market availability, clinical implementation, reimbursement policies and pharmacy practice related to CBD and cannabinoid-based products.

### 2.1. Regulatory Approaches and Clinical Implementation of CBD in Selected European Countries

Of the European countries, the analysis included Germany, the Netherlands, Slovenia, Italy, France, Finland and Croatia. For each analysed country, information on the legislative framework and the availability of CBD-containing products is provided.

#### 2.1.1. Germany

Germany has adopted one of the more liberal cannabis policy approaches in Europe. Medical cannabis has been legal since 2017, while the introduction of the Cannabis Act in 2024 further expanded access by legalising the possession and limited cultivation of cannabis for recreational purposes [34]. These regulatory developments reflect the growing diversification of cannabis-derived products, including CBD-containing products, on the German market. Following the entry into force of the Cannabis Act on 1 April 2024, cannabis intended for medical and scientific purposes was removed from the scope of the German Narcotics Act (BtMG) and is now regulated separately under the Medical Cannabis Act (MedCanG) [35]. Epidyolex^®^ is currently the only authorised medicinal product containing purified CBD in Germany. In addition, Sativex^®^ (nabiximols), containing THC and CBD in an approximately 1:1 ratio, is approved for the treatment of moderate to severe spasticity associated with multiple sclerosis. Nevertheless, CBD-containing products, including oils, capsules, cosmetics, and e-liquids, are widely available on the market. The legal status of these products depends not only on their THC content (legal if they contain less than 0.3% THC) but also on their intended use and regulatory classification, including whether they are marketed as medicinal products, cosmetics, or food products [36]. According to the German Federal Institute for Risk Assessment (BfR), CBD-containing foods and food supplements are currently not authorised on the German market. In addition, the novel food assessment process for CBD products remains suspended because of insufficient safety data [36]. Under the European Union Novel Food Regulation, foods and food ingredients that were not consumed to a significant extent within the EU before 15 May 1997 must undergo a safety assessment and obtain authorisation before being placed on the market [37]. According to the European Commission Novel Food Catalogue, extracts of *Cannabis sativa* L. and cannabinoid-containing products, including CBD, are classified as novel foods. This includes cannabinoid extracts, foods containing such extracts, and synthetically produced cannabinoids [33,38]. This distinction between authorised medicinal products and non-authorised CBD-containing products is clinically important because it determines the level of regulatory oversight, available evidence regarding safety and efficacy, and the extent of healthcare professional involvement in patient care. Clinical implementation of cannabinoid-based medicines in Germany is considerably more advanced than in many other European countries. Since the legalisation of medical cannabis in 2017, physicians have been able to prescribe cannabis flowers and cannabis-based medicinal products for patients with serious illnesses when conventional therapies are not considered adequate. Cannabinoid-based therapies are most commonly used in the management of chronic pain, spasticity associated with multiple sclerosis, chemotherapy-induced nausea and vomiting, and certain forms of treatment-resistant epilepsy. In addition to approved medicinal products such as Epidyolex^®^, magistral preparations and medical cannabis flowers are available through pharmacies upon prescription. In selected cases, reimbursement may be provided by health insurance following prior approval [35,39]. The German model therefore combines broad availability with physician-directed prescribing and reimbursement mechanisms, allowing treatment decisions to be based on individual clinical assessment, expected therapeutic benefit and consideration of potential risks.

Pharmacists play a central role in the implementation of cannabinoid therapies in Germany. Community and hospital pharmacists are responsible for dispensing cannabis-based medicinal products, preparing magistral cannabis formulations, verifying prescription appropriateness and ensuring product quality and traceability. Pharmacists are involved in the preparation of magistral cannabis formulations according to standardised DAC/NRF procedures and quality standards [40]. They also play a key role in ensuring the safe and appropriate use of cannabinoid-based medicines through patient counselling, therapeutic monitoring and adverse-effect management. Continued training of healthcare professionals remains essential to support evidence-based prescribing and optimise clinical outcomes [31,41]. Since the entry into force of the German Cannabis Act (CanG) on 1 April 2024, adults aged 18 years and older are permitted to possess up to 25 g of dried cannabis in public and up to 50 g of dried cannabis at their place of residence for personal use. In addition, adults may cultivate up to three cannabis plants for personal consumption [39]. These changes primarily concern non-medical cannabis use and should be distinguished from the medically supervised use of cannabinoid-based therapies, which remains dependent on prescription pathways, quality standards and clinical evaluation.

#### 2.1.2. Netherlands

The Netherlands is another European country with a relatively liberal approach to cannabis, characterised by a long-standing “tolerance policy”. Under this system, the possession and sale of small quantities of cannabis are formally illegal but tolerated under specific conditions [42]. In the Netherlands, the Office of Medicinal Cannabis (OMC) is responsible for regulating medical cannabis, including its production, quality control, distribution, import, and export [15]. Cannabis producers must be clicensed by the Dutch government and are required to sell all their production to OMC, which then distributes it to pharmacies. This centrally regulated system aims to ensure consistency in product quality and cannabinoid composition, both of which are essential for the safe clinical use of cannabinoid-based therapies [15,43]. The composition varies in THC and CBD content, and cannabis can be prescribed for cancer pain, multiple sclerosis, HIV, chronic neurological pain, and tics associated with Tourette syndrome [28]. The Dutch medical cannabis system includes several standardised cannabis products that are available through pharmacies. Currently, five different medical cannabis products are available in the Netherlands: Bedrobinol, Bedrocan, Bediol, Bedrolite, and Bedica. These products differ in their tetrahydrocannabinol (THC) and cannabidiol (CBD) concentrations [43]. The main characteristics and cannabinoid composition of medical cannabis products available through Dutch pharmacies are presented in Table 2.

In addition to medical cannabis products available by prescription, CBD oil preparations containing either CBD alone or CBD in combination with THC may be compounded and dispensed through pharmacies. Furthermore, numerous CBD-containing products are widely available through online platforms and retail outlets, including health food stores and drugstores, where they are typically marketed as dietary supplements or wellness-related products rather than authorised medicines [37,44,45]. The coexistence of pharmacy-dispensed medicinal cannabis and commercially available consumer CBD products is clinically relevant because the latter are not evaluated according to the same pharmaceutical standards for quality, safety, and efficacy [46]. In the Netherlands, CBD products are generally tolerated if their THC content does not exceed 0.05%, a threshold that is among the most restrictive for CBD products in Europe [46]. The Netherlands Food and Consumer Product Safety Authority (NVWA) is responsible for overseeing CBD-containing consumer products and enforcing the provisions of the EU Novel Food Regulation applicable to orally consumed CBD products [32]. Regulatory oversight of non-medicinal CBD products is essential to ensure product quality and patient safety [32]. Although the Netherlands has a well-established medicinal cannabis programme, patient access may be limited by the lack of routine reimbursement [44]. In contrast, eligible patients in Germany may receive reimbursement through statutory health insurance, potentially improving treatment accessibility and continuity [39].

#### 2.1.3. Slovenia

In 2025, Slovenia adopted the Act on Cannabis for Medical and Scientific Purposes (ZKMZN), establishing a specific legal framework governing the production, trade, import, export, and use of cannabis for medical and scientific purposes [47]. The law defines medical cannabis broadly, including cannabis plant material, extracts, preparations, and naturally derived cannabinoids regardless of THC content [48]. The new legislation establishes a structured framework for the clinical use of cannabinoid-based therapies, although its successful implementation will require evidence-based clinical guidance and appropriate professional education [47,49]. Access to cannabinoid-based medicines in Slovenia is regulated through approved medicinal products, temporary import procedures, and magistral preparations prescribed by physicians. Epidyolex, a cannabidiol (CBD) containing medicinal product used for specific forms of epilepsy, has been authorised in all EU Member States, including Slovenia, since 2019. In addition, Slovenia permits access to other cannabinoid-based medicines, including Sativex, through temporary import procedures under specific medical conditions. Slovenian legislation also allows magistral cannabinoid preparations containing tetrahydrocannabinol (THC), cannabidiol (CBD), or their combination to be prescribed and prepared in pharmacies under medical supervision [31,50].

As in many other EU countries, CBD products intended for oral consumption in Slovenia are regulated under the EU Novel Food framework and are currently classified as unauthorised novel foods, including products containing added cannabinoids such as CBD-enriched hemp oils. According to the European Food Safety Authority (EFSA), the safety of CBD as a novel food has not yet been established because of insufficient toxicological and safety data. Furthermore, health claims related to CBD-containing food products are currently not authorised, and CBD products without medicinal marketing authorisation may not be marketed or advertised as having therapeutic or disease-preventing properties [51]. The legislative reforms introduced in 2025 are expected to strengthen the clinical integration of cannabinoid-based therapies into Slovenian healthcare practice by expanding access to authorised medicines, magistral preparations, and imported cannabinoid products [47,48,51]. However, the successful implementation of this legislation will require evidence-based clinical guidance, continued training of healthcare professionals, and ongoing evaluation of treatment outcomes and safety [49].

#### 2.1.4. Italy

In 2025, Italy introduced Decree-Law No. 48/2025, restricting the possession and sale of industrial hemp flowers and bringing them within the scope of narcotics regulation. Under the new legislation, industrial hemp flowers were brought within the scope of narcotics regulation regardless of THC concentration, and strict restrictions were introduced on activities involving hemp flowers and certain hemp-derived cannabinoids. CBD for oral use is currently limited to prescription pharmaceutical purposes under the Decree of the Health Ministry of 27 June 2024 [52,53,54]. However, concerns have been raised regarding the compatibility of these restrictions with European Union law and with previous decisions of the Court of Justice of the European Union on the marketing of CBD products within the EU [53]. In 2020, the Court of Justice of the European Union (CJEU) ruled that Member States may not prohibit the marketing of CBD that has been legally produced in another Member State, unless legitimate public health concerns justify such restrictions and are proportionate to the objective pursued [53,55]. Cannabis-based therapies available in Italy include authorised medicinal products such as Epidyolex^®^ and Sativex^®^, magistral cannabis preparations, and cannabinoid preparations containing dronabinol or nabilone prescribed under specific medical conditions. They may be prepared in specialised pharmacies [56]. Medical cannabis used for magistral preparations may originate from domestic production or authorised imports [53,57]. This medically supervised model facilitates the integration of cannabinoid-based therapies into clinical practice through defined prescribing pathways and pharmacy-based compounding, although access remains restricted to specific indications [56,58]. As in Germany and the Netherlands, pharmacists in Italy play a key role in dispensing cannabinoid-based medicines and preparing magistral cannabis formulations, thereby supporting the safe and effective use of cannabis-based therapies in clinical practice [56,57]. Despite the established medical cannabis framework, continued clinical guidance and professional education remain essential for evidence-based prescribing [49].

#### 2.1.5. France

When discussing French CBD regulations, the Kanavape case represents an important milestone. In 2020, the Court of Justice of the European Union (CJEU) ruled that cannabidiol (CBD) legally produced from the whole *Cannabis sativa* L. plant could not be regarded as a narcotic drug within the meaning of the 1961 United Nations Single Convention on Narcotic Drugs, provided that it does not have psychotropic effects [28]. France has historically maintained a restrictive approach toward cannabis and CBD products. In 2021, the French government introduced a ban on the sale of CBD-rich cannabis flowers and leaves. However, the measure was subsequently suspended by the Council of State (Conseil d’État), which ruled that a general prohibition was disproportionate in light of the available scientific evidence regarding the risks associated with CBD [28]. According to current French regulations, hemp extracts and CBD-containing products are permitted provided that they are derived from authorised hemp varieties and contain no more than 0.3% THC. Since 2015, CBD has become widely available in a variety of consumer products, including oils, cosmetics, e-liquids, capsules, confectionery products, and hemp flowers [28,59].

A national French survey reported that approximately 10% of adults had used CBD and identified several user profiles. The authors concluded that clearer European regulations are needed to ensure the availability of safe and high-quality CBD products [60].

Regarding cannabinoid-based medicines in France, Epidyolex^®^ and Sativex^®^ are among the few cannabis-derived medicinal products authorised or accessible within the French medical cannabis framework. Their use is generally restricted to specific clinical indications and requires prescription and medical supervision [59,61]. This restricted access reflects the current emphasis on controlled clinical use, where treatment decisions are based on medical evaluation rather than general availability of CBD products. Certain non-medicinal CBD products are available in pharmacies, provided they meet the requirements applicable to their regulatory category [59].

Over-the-counter CBD-containing products, including oils, capsules, cosmetics, and other wellness preparations, are widely available in France, including through community pharmacies. Pharmacists play an important role in counselling patients on the appropriate use of these products, potential adverse effects and drug interactions, and in promoting their safe and evidence-based use [61].

#### 2.1.6. Finland

Finland represents a Nordic conservative regulatory model characterised by a strict regulatory framework and strong quality-control standards for CBD products [62].

Finland has adopted a relatively restrictive regulatory approach toward CBD products. According to the Finnish Medicines Agency (Fimea), preparations containing cannabidiol (CBD) are generally classified as medicinal products and are therefore subject to medicinal product legislation. Fimea assesses products on a case-by-case basis to determine their regulatory status. Consequently, the importation of CBD-containing products without a valid prescription may be prohibited depending on the product composition and country of origin. Furthermore, cannabinoid extracts and food products containing CBD are classified as novel foods under European Union legislation and may not be marketed without prior authorisation. Such products are also prohibited from making medicinal or health claims [62]. Clinical implementation of cannabinoid-based therapies in Finland remains limited and is primarily restricted to authorised medicinal products such as Sativex^®^ and Epidyolex^®^, while access to herbal medical cannabis is possible only through a special permit procedure administered by Fimea. Pharmacy compounding of cannabinoid-based magistral preparations does not appear to be routinely implemented in Finland [62]. From a patient safety perspective, Finland’s restrictive regulatory framework limits access to inadequately characterised cannabis-based products, with non-authorised preparations available only under Fimea’s special-permit procedure [62]. Such a restrictive approach evidently limits the clinical application of cannabinoid-based therapies.

#### 2.1.7. Croatia

Croatia generally follows the European Union regulatory framework governing CBD-containing products. CBD-containing medicinal products are regulated under pharmaceutical legislation, while non-medical CBD products are subject to EU novel food and consumer product regulations [27,33,37]. Currently, Epidyolex^®^ is the only CBD-based medicinal product authorised in Croatia through the centralised procedure of the European Medicines Agency [27]. Reimbursement of Epidyolex^®^ is restricted to approved indications and specific reimbursement criteria defined by the Croatian Health Insurance Fund (HZZO). Reimbursement is available for patients aged ≥ 2 years with Lennox–Gastaut syndrome, Dravet syndrome (in combination with clobazam), or tuberous sclerosis complex whose seizures remain inadequately controlled despite treatment with at least two appropriate antiepileptic drugs. Treatment initiation requires approval by the hospital medicines committee based on the recommendation of an epilepsy specialist, and continuation of reimbursement depends on regular assessment of treatment response [63]. In Croatia, medicinal products containing dronabinol, THC, or nabilone may be prescribed by general practitioners following the recommendation of authorised medical specialists, including neurologists, oncologists, infectologists, and neuropediatricians, for selected indications such as multiple sclerosis, cancer-related symptoms, treatment-resistant epilepsy, and HIV/AIDS, in accordance with national regulations [64]. The Croatian CBD market has expanded considerably in recent years, and CBD oils, capsules, cosmetics, and other hemp-derived products are widely available through pharmacies, specialised stores, and online platforms. Strengthening the role of pharmacists in patient counselling may be particularly valuable given their accessibility and frequent contact with patients seeking non-prescription CBD products [65]. The expanding Croatian market for CBD-containing products also highlights the importance of effective market oversight to ensure product quality, accurate labelling, consumer safety, and compliance with national and European regulatory requirements. Studies conducted in Croatia have demonstrated generally positive attitudes toward CBD among physicians, pharmacists, and healthcare students, while simultaneously identifying substantial educational gaps regarding its therapeutic applications, safety profile, and regulatory status. More than 80% of respondents reported a need for additional education on CBD, highlighting the importance of improving cannabinoid-related knowledge among healthcare professionals. The current role of community pharmacists in cannabinoid-based therapy remains limited owing to the restricted integration of cannabinoid-based therapies into routine clinical practice, educational gaps, and limited opportunities for professional involvement. Regulatory uncertainty, limited clinical guidelines, and insufficient professional training remain important barriers to the broader implementation of CBD in healthcare practice in Croatia. In addition, the persistent association of CBD with recreational cannabis may contribute to stigma among both healthcare professionals and patients [66]. At the same time, Croatian researchers have contributed to the growing evidence base on CBD. Clinical studies conducted in patients with hypertension have provided preliminary data on CBD pharmacokinetics, tolerability, and potential cardiovascular effects; however, further controlled clinical studies are required [25,26,67].

Croatia represents a market with broad availability of CBD-containing products, while the clinical integration of CBD into routine healthcare practice remains limited. Future efforts should therefore focus on developing evidence-based clinical recommendations, improving professional education and establishing clearer pathways for the safe implementation of cannabinoid-based therapies within Croatian healthcare practice. The regulatory status, consumer availability, clinical access, reimbursement, pharmacist involvement, and key implementation barriers across Croatia and the selected countries are summarised in Table 3 and Table 4.

## 3. Discussion

Overall, differences in national regulatory frameworks extend beyond legal classification and have important implications for the clinical implementation of cannabinoid-based therapies. Across the analysed countries, regulatory approaches influence patient access, prescribing pathways, reimbursement, pharmacist involvement, patient counselling, and the integration of cannabinoid-based medicines into evidence-based clinical practice. Germany and the Netherlands have well-developed systems for the medical use of cannabis. Nevertheless, Germany places greater emphasis on reimbursement through statutory health insurance and formal approval procedures, whereas in the Netherlands patients more commonly bear the costs of treatment themselves. Germany has recently adopted a formal legalisation framework through the Cannabis Act [34,35,42]. In contrast, the Dutch model is traditionally based on a policy of tolerance combined with a centrally regulated medical cannabis programme administered by the Office of Medicinal Cannabis. Germany has established domestic production of medical cannabis under the supervision of BfArM; many medical cannabis products continue to be imported from other countries, particularly the Netherlands, Canada, and Portugal [35].

Compared with Germany and the Netherlands, Slovenia has only recently established a comprehensive legal framework for medical cannabis, and the practical implementation of the new legislation is still evolving [48,51]. Successful integration of the new regulatory framework into clinical practice will require clear prescribing pathways, continued education of physicians and pharmacists, and evidence-based clinical guidance.

Compared with Germany and the Netherlands, Italy demonstrates a more restrictive approach toward CBD products despite maintaining a well-established system for the prescription, preparation, and dispensing of medical cannabis [53,57].

France and Italy represent different approaches to cannabinoid regulation. While Italy has focused primarily on the medical use of cannabis through authorised medicines and magistral preparations, France has developed a broader consumer CBD market. However, the clinical implementation of cannabinoid-based therapies remains more limited [56,59,68]. The coexistence of authorised medicinal products and widely available consumer CBD products highlights the importance of patient counselling and clear differentiation between evidence-based medical treatment and non-medical CBD use [69].

Finland represents one of the most restrictive approaches among the countries analysed, with strict regulation of both CBD medicines and CBD-containing consumer products, resulting in limited market availability and clinical use compared with several other European countries [62]. Although this approach may strengthen quality assurance and patient safety, it may also reduce timely access to cannabinoid-based therapies for patients who could potentially benefit from treatment.

The implementation of CBD and cannabinoid-related prescription medicines into routine clinical practice remains heterogeneous across European countries and depends on healthcare systems, reimbursement policies, and professional education, although regulatory pathways for these medications have become more clearly defined [31].

Several European studies, including those from Croatia, have highlighted the need for clearer clinical guidance and improved training of healthcare professionals regarding cannabinoid-based therapies. Education should focus on evidence-based prescribing, dose titration, safety, clinically relevant drug interactions, and patient counselling. Strengthening the role of pharmacists within multidisciplinary healthcare teams may further improve medication safety and therapeutic outcomes [49,61,66]. Compared with Germany, the Netherlands, and Canada, the integration of cannabinoid-based therapies into routine clinical practice remains more limited in Croatia, with pharmacists playing a relatively minor role in cannabinoid-related patient care [64,66]. Similar barriers have been reported in other European countries and include limited professional training, insufficient clinical guidance, and restricted practical experience among healthcare professionals [31,60,66]. The incorporation of cannabinoid-related topics into healthcare curricula, together with the development of comprehensive clinical guidelines similar to those available in Canada, may improve professional knowledge and facilitate the evidence-based integration of cannabinoid-based therapies into clinical practice.

From a global perspective, Canada is widely regarded as one of the most liberal countries in the world regarding cannabis policy. The Cannabis Act, enacted in 2018, legalised both medical and non-medical cannabis use and created a comprehensive national system for cannabis production, distribution, prescribing, and patient access [70]. The Canadian experience demonstrates that successful clinical implementation depends not only on legal access but also on structured prescribing frameworks, reimbursement mechanisms, multidisciplinary collaboration, and the availability of evidence-based clinical guidance [71]. Prescription medications like nabiximols (Sativex^®^), cannabidiol (Epidiolex^®^), and nabilone are available within the healthcare system; qualified healthcare professionals, such as physicians and nurse practitioners, are authorised to prescribe medical cannabis for a variety of conditions [71]. Reimbursement varies between provinces and indications, with some cannabinoid-based medicines covered by provincial healthcare systems, whereas medical cannabis is more commonly reimbursed through private insurance or specific federal programmes [70,71]. Canada has also developed extensive clinical guidance for healthcare professionals and patients, supporting evidence-based prescribing, monitoring, and risk management [72].

Interestingly, despite being one of the most liberal countries regarding medical and recreational cannabis, Canada relies primarily on federally licenced manufacturers for the supply of medical cannabis, while pharmacist-led compounding of cannabis-containing preparations remains restricted and is prohibited in some provinces [73]. In contrast, pharmacists in Germany, the Netherlands, and Slovenia play a central role in the preparation and dispensing of magistral cannabis formulations. Australia represents another example of a country with a well-developed regulatory framework for medical cannabis. Since the legalisation of medical cannabis in 2016, patient access has expanded substantially through the Special Access Scheme and Authorised Prescriber pathways administered by the Therapeutic Goods Administration (TGA) [74].

Similar to Canada, Australia has developed comprehensive clinical guidance and educational resources to support healthcare professionals involved in cannabinoid-based therapies. Despite the increasing availability of medical cannabis products, most cannabinoid-based medicines remain unregistered and are supplied through special access pathways rather than standard marketing authorisation procedures [74,75]. Interestingly, although Australia introduced a regulatory pathway for pharmacist-only low-dose CBD products in 2021, no such product has yet been approved for routine over-the-counter supply, illustrating a gap between regulatory reform and practical implementation that remains evident in many European countries [74,75]. In Australia, reimbursement is product-specific. Epidyolex^®^ has been listed on the Pharmaceutical Benefits Scheme (PBS) since May 2021 for eligible patients who meet specified authority criteria, whereas most other medicinal cannabis products are not PBS-subsidised and are paid for by patients [76].

These findings indicate that regulatory frameworks alone do not ensure successful clinical implementation, which is also shaped by reimbursement, professional training, clinical guidance, pharmacist involvement, and patient safety considerations. This is particularly relevant to Croatia, where improved training and clearer guidance may support broader integration into routine healthcare practice.

## 4. Materials and Methods

### Literature Search Strategy

This narrative review was designed to analyse the regulation, market availability, and clinical use of cannabidiol (CBD) in Croatia in relation to selected European countries, Canada, and Australia. Literature searches were performed between May and June 2026 using the PubMed, Scopus, and Web of Science databases. Additional information was obtained from official regulatory and governmental sources, including the European Medicines Agency (EMA), European Food Safety Authority (EFSA), European Commission, European Union Drugs Agency (EUDA), Croatian Agency for Medicinal Products and Medical Devices (HALMED), and national regulatory authorities of the selected countries. Reference lists of relevant reviews, clinical guidelines, and regulatory documents were manually screened to identify additional relevant sources.

The search strategy included combinations of the terms “cannabidiol”, “CBD”, “cannabinoids”, “medical cannabis”, “regulation”, “clinical practice”, “pharmacy practice”, “dietary supplements”, “novel food”, “training for pharmacists”, “European Union”, and “Croatia”. Search terms and combinations were adapted to the structure and indexing system of each database.

Priority was given to literature published within the last ten years, although older studies and key regulatory documents were included when relevant. Scientific literature published in English and official regulatory documents available in their original language were included. References addressing CBD regulation, market availability, clinical use, pharmacy practice and relevant legislative aspects were selected according to their relevance to the objectives of this review, whereas publications unrelated to these objectives or focusing on basic experimental research without regulatory or clinical relevance were excluded. Priority was given to regulatory documents, clinical guidelines, systematic reviews, meta-analyses, randomised controlled trials, and observational studies, where available. Regulatory documents and official publications issued by national and international authorities were considered the primary sources for the regulatory analysis and were complemented by relevant scientific literature. The analysis included Croatia, Germany, the Netherlands, Slovenia, Italy, France, and Finland, with Canada and Australia discussed as international reference models. As this was a narrative review, no PRISMA flow diagram, formal risk-of-bias assessment, or meta-analysis was performed. Evidence was interpreted considering study design, methodological quality, regulatory relevance, and consistency across studies. Regulatory information and official documents were reviewed up to 3 August 2026.

## 5. Conclusions

Current evidence indicates significant variation in regulatory frameworks, patient access, product availability, and the integration of cannabinoid-based therapies into healthcare systems across Croatia, the selected European countries, Canada and Australia. Within the European Union, regulation and clinical implementation remain largely under national jurisdiction, resulting in differences in patient access, prescribing practices, reimbursement policies and the role of pharmacists. These regulatory differences have important clinical implications, influencing the availability of evidence-based cannabinoid therapies, healthcare professional involvement and the consistency of patient care.

Future efforts should focus on strengthening the clinical implementation of cannabinoid-based therapies through the development of evidence-based clinical guidelines, improved education of healthcare professionals, appropriate patient counselling, multidisciplinary collaboration between physicians and pharmacists and continued harmonisation of regulatory frameworks. Continued attention should also be given to improving the quality, traceability and safety of CBD-containing products. Continued efforts are also needed to improve the quality, traceability and safety of CBD-containing products through traceable supply chains, certificates of analysis, regular monitoring of contaminants, pesticides, heavy metals and additives, alongside compliance with national and European regulatory requirements.

Future research should expand beyond CBD and THC to evaluate the therapeutic potential of minor cannabinoids and their possible synergistic interactions. Clinical studies are also needed to further clarify efficacy, long-term safety, optimal dosing strategies and appropriate patient selection across different clinical indications. Despite the growing availability of CBD-containing products in Croatia, the clinical implementation of cannabinoid-based therapies remains limited. Improved training of healthcare professionals, particularly physicians and pharmacists, and clearer clinical guidance may facilitate their evidence-based integration into clinical practice in Croatia and other European countries where implementation remains limited.

## Figures and Tables

**Table 1:** Definitions of key cannabis- and CBD-related terms used throughout this review.

Term	Definition
CBD consumer products	Non-medicinal products containing cannabidiol (CBD) for consumer use.
CBD food supplements	CBD consumer products intended for oral consumption and marketed as food supplements.
Novel food	Regulatory status of CBD used in foods and food supplements under the European Union Novel Food Regulation.
CBD medicinal products	CBD-containing medicinal products granted marketing authorisation (e.g., Epidyolex^®^ in the European Union, marketed as Epidiolex^®^ in the United States *).
Cannabinoid-based medicines	Medicinal products containing cannabinoids, including authorised products and products available through national regulatory pathways, such as magistral preparations.
Medical cannabis (also referred to as medicinal cannabis)	Cannabis used for medical purposes under healthcare-professional supervision.
Recreational cannabis (non-medical cannabis)	Cannabis used outside recognised medical indications and medical supervision.

* Definitions were adapted from the European Commission Novel Food Catalogue and EMA product information for Epidyolex [27,33].

**Table 2:** Types of medical cannabis in the Netherlands; product name, THC and cannabidiol content.

Type	% Tetrahydrocannabinol (THC)	% Cannabidiol (CBD)
Bedrobinol^®^	~13.5	<1
Bedrocan^®^	~22	<1
Bediol^®^	~6.3	~8
Bedica^®^	~14	<1
Bedrolite^®^	<1	~7.5

**Table 3:** Consumer CBD regulation and availability in Croatia and the selected countries.

Country	Consumer CBD	Food/FS (NF) Status	Cosmetics	Key National Feature
Croatia	Widespread	NF authorisation required	Permitted under EU cosmetics rules	Limited clinical implementation
Germany	Widespread	NF authorisation required	Permitted under EU cosmetics rules	Consumer CBD separate from MC under MedCanG
The Netherlands	Widespread (≤0.05% THC)	NF authorisation required	Permitted under EU cosmetics rules	OMC programme; compounded CBD oils
Slovenia	Restricted (oral)	NF authorisation required	Permitted under EU cosmetics rules	2025 Act for medical/scientific cannabis
Italy	Limited (oral)	NF authorisation required; national oral-CBD restrictions	EU cosmetics rules + national restrictions	Restrictive, evolving oral-CBD framework
France	Widespread	NF authorisation required	Permitted under EU cosmetics rules	Hemp-derived CBD permitted (≤0.3% THC)
Finland	Restricted	NF authorisation required; possible medicinal classification	EU cosmetics rules; claims/composition may trigger medicinal classification	Case-by-case medicinal classification
Canada	Widespread via authorised channels	Cannabis Act; not a conventional FS	Regulated as cannabis topicals	CBD regulated as cannabis
Australia	Restricted; no approved low-dose CBD products	Not a conventional FS; therapeutic CBD is a medicine	Non-therapeutic cosmetics permitted; therapeutic claims trigger TGA regulation	Access relies largely on special-access pathways for unregistered cannabinoid products

Note: In EU countries, CBD extracts and isolates used in foods/FS are subject to NF authorisation; cosmetics are governed by Regulation (EC) No 1223/2009 and national enforcement. Abbreviations: CBD, cannabidiol; EU, European Union; FS, food supplement(s); MC, medical cannabis; MedCanG, Medical Cannabis Act; NF, Novel Food; OMC, Office of Medicinal Cannabis; THC, delta-9-tetrahydrocannabinol. Consumer CBD availability refers to practical market presence and does not imply legal authorisation as a food, food supplement, cosmetic product, or medicinal product. “Widespread” indicates broad practical availability through retail, pharmacy, or online channels; “restricted” indicates that availability is constrained by national classification, route-specific requirements, or enforcement practices; and “limited” indicates a narrow practical market presence.

**Table 4:** Clinical access and implementation of cannabinoid-based therapies in Croatia and the selected countries.

Country	Authorised CBD/CBM	MC/CBM Access	Reimbursement	Pharmacist Role	Main Barriers
Croatia	Epidyolex	Specialist recommendation; limited THC/dronabinol/nabilone access	Product/indication-specific	Limited clinical involvement	Availability; unclear pathways; limited guidance
Germany	Epidyolex; nabiximols	Prescription; medicines, flowers, extracts and magistral preparations	SHI coverage subject to requirements	Primary role; compounding	Administration; cost; evidence gaps
The Netherlands	Epidyolex; nabiximols	Prescription; OMC products and compounded CBD oils	Epidyolex conditional; MC usually not routine	Primary role; compounding	Limited reimbursement; evidence gaps
Slovenia	Epidyolex; other authorised products if marketed	Authorised products, temporary import and magistral preparations	Product-specific	Primary role; compounding	Early implementation; availability; limited guidance
Italy	Epidyolex; nabiximols	Prescription; state production/import and magistral cannabis	Region-dependent	Primary role; compounding	Regional variation; supply; evolving oral-CBD rules
France	Epidyolex; nabiximols	Authorised medicines; broader MC access limited	Product/indication-specific	Primary role	Limited access; reimbursement uncertainty
Finland	Epidyolex; nabiximols	Prescription; Fimea special permit for unauthorised products	Limited/case-specific	Primary role; permit submission	Classification; permits; prescriber experience
Canada	Epidiolex; Sativex; nabilone	Prescription medicines; separate MC pathway	Variable public/private coverage	Counselling/medication review; limited distribution role	Fragmented reimbursement; separate pathway
Australia	Epidyolex	SAS/AP; most products unregistered	Epidyolex PBS-listed; others mostly self-paid	Dispensing and counselling; low-dose CBD pathway not yet available in practice	Unregistered products; cost; variable access

Note: Authorisation does not imply market availability or reimbursement. Core pharmacist activities include dispensing, counselling, interaction screening and PV; only country-specific additions or limitations are shown. Abbreviations: AP, Authorised Prescriber; CBD, cannabidiol; CBM, cannabinoid-based medicine(s); MC, medical cannabis; OMC, Office of Medicinal Cannabis; PBS, Pharmaceutical Benefits Scheme; PV, pharmacovigilance; SAS, Special Access Scheme; SHI, statutory health insurance; THC, delta-9-tetrahydrocannabinol. “Authorised” refers to formal marketing authorisation as a medicinal product and does not necessarily imply national market availability, reimbursement, or routine clinical use. Prescription access refers to products available through standard prescribing pathways, whereas special access refers to named-patient, temporary-import, special-permit, Special Access Scheme, or Authorised Prescriber pathways. “Limited access” indicates that use is restricted to selected products, indications, prescribers, or access procedures. Reimbursement is reported separately and refers only to public or insurance coverage under specified eligibility criteria. The primary pharmacist role includes dispensing, patient counselling, screening for clinically relevant drug interactions, and pharmacovigilance. Country-specific additional activities or limitations, such as compounding, permit submission, medication review, or restricted distribution, are indicated separately where applicable.

## Data Availability

The data presented in this study are available upon request from the corresponding author.
